# Various aspects of upper-limb muscle strength and tennis performance: A comparative study of U12 and U14 female tennis players

**DOI:** 10.1371/journal.pone.0362183

**Published:** 2026-11-06

**Authors:** Tomasz Waldziński, Aleksandra Durzyńska, Bartłomiej Niespodziński, Jan Mieszkowski, Ewa Waldzińska, Andrzej Kochanowicz

**Affiliations:** 1 Faculty of Health Sciences, University of Lomza, Łomża, Poland; 2 Faculty of Health Sciences and Physical Education, Kazimierz Wielki University, Bydgoszcz, Poland; 3 Department of Gymnastics and Dance, Gdańsk University of Physical Education and Sport, Gdańsk, Poland; University of Belgrade: Univerzitet u Beogradu, SERBIA

## Abstract

This study investigated the relationship between muscle strength and tennis performance, assessed using the International Tennis Number (ITN) on-court test, operationalized as point-based outcomes (total points and subcomponent points), in young female tennis players aged under 12 (U12) and under 14 (U14). Thirty-three players underwent a comprehensive evaluation including ITN testing, general strength tests (handgrip strength, flexed arm hang, and tennis-ball throw), isometric upper-limb muscle strength, and sense of force assessment. Results demonstrated significantly higher ITN scores and muscle strength in the U14 group, with differences largely attributable to body size and training experience. Correlation analyses revealed age-specific patterns: in U12 players, ITN scores were associated primarily with general strength measures, whereas in U14 players, they were more strongly linked to joint-specific upper-limb strength and neuromuscular characteristics. Sense of force did not differ between groups, though correlations with ITN varied depending on age and joint function. These findings suggest that, in this sample, performance in younger players was more closely associated with general strength measures, whereas in older players it was more closely associated with joint-specific upper-limb strength.

## Introduction

Tennis performance is determined by multiple technical, tactical, physical, and developmental factors [1–3]. Among the physical capacities involved in tennis performance, muscle strength represents an important neuromuscular component contributing to stroke production, movement efficiency, postural stability, and joint control [4,5]. Greater strength facilitates force generation and transmission throughout the kinetic chain and support the execution of repeated tennis-specific actions. In this context, upper-limb strength may be particularly relevant to racket control and force transmission during stroke production, thereby contributing to the effective execution of tennis-specific technical tasks.

Previous research showed that muscle strength is associated with tennis performance. For instance, players achieving higher performance levels have demonstrated greater explosive strength, reflected in associations with vertical jump performance and overhead medicine-ball throw distance [1]. Athletes with greater lower-limb explosive strength have also shown superior performance in speed tests and higher ball velocities during the serve [6]. Likewise, greater trunk rotational strength has been associated with improved stroke stabilization, which may contribute to shot precision and directional control [7]. However, although the contribution of general and lower-limb strength to tennis performance has been investigated relatively extensively, considerably less is known about the relationship between upper-limb strength characteristics and on-court performance, particularly in young female players. Understanding these associations may provide further insight into the neuromuscular characteristics accompanying tennis performance during junior development.

The development of muscle strength undergoes marked changes throughout ontogeny, reflecting both biological maturation and adaptations to external stimuli [8,9]. Although partly determined by genetic factors, strength development is strongly influenced by environmental factors, particularly systematic sports training [10]. In girls, the development of strength capacities is especially pronounced between the ages of 10 and 14 years [11,12]. During this maturational period, substantial changes occur in muscle mass and neuromuscular coordination, providing the basis for improvements across different manifestations of muscle strength. A two-year longitudinal study demonstrated that knee-extensor strength in elite young tennis players increases markedly after the age of 13, particularly during high-velocity movements [13]. Similarly, force-sense development in young female tennis players appears to approach the level observed in older age groups around 12–13 years of age [14]. After this critical period, the rate of muscle-strength development in girls slows considerably [9,15,16]. These developmental characteristics provide a rationale for comparing under-12 (U12) and under-14 (U14) players. Within this developmental framework, a standardized assessment of on-court performance is needed to examine how different strength characteristics relate to tennis performance. The International Tennis Number (ITN) on-court test provides such a framework, incorporating tennis-specific tasks involving stroke depth, accuracy, serve execution, and mobility [1]. In addition to providing an overall point-based score, the ITN allows performance to be examined across individual subcomponents, making it particularly suitable for investigating associations between specific strength characteristics and different aspects of on-court performance. Therefore, the aim of the present study was twofold: (i) to compare multiple aspects of muscle strength between U12 and U14 female tennis players, and (ii) to evaluate the relationship between ITN point-based outcomes and various strength characteristics within these groups.

## Methods

### Study design

This cross-sectional study involved two groups of female tennis players U12 and U14 —who underwent a comprehensive assessment of tennis performance and selected indices of muscle strength. Performance was evaluated using the ITN total points and ITN subcomponent points derived from the ITN on-court test, while general muscle strength was examined through a handgrip strength test (HGS), a flexed arm hang test (FAH), and a tennis ball throw for distance test (TBT). In addition, isometric muscle strength and sense of force of the dominant upper limb were assessed.

All procedures were conducted over two consecutive days in the Exercise Physiology Laboratory of the Gdańsk University of Physical Education and Sport, Gdańsk, Poland. Additionally, a subsample returned for a second ITN session for test–retest reliability assessment. Prior to testing, participants were familiarized with all procedures and, on both testing days, completed a standardized warm-up to optimize performance.

On day 1, participants initially completed the isometric muscle strength assessment of the dominant upper limb. Following a one-hour recovery period, the sense of force test was administered to evaluate the accuracy of muscle strength perception and control.

On day 2, the ITN test was administered to assess overall tennis performance. After a three-hour rest interval, participants completed, in sequence: TBT test, HGS test, and FAH test. Short passive rest periods were provided between trials to ensure consistent and repeatable performance.

### Participants

Recruitment took place between 16 November 2020 and 23 April 2021. A total of 33 female tennis players were enrolled and stratified into two age categories: U12 and U14. Players were selected by their coaches from seven regional tennis clubs as the most talented athletes and were recruited on the basis of their tennis skills assessed by the ITN level, with a minimum ITN level of 8 for U12 and 6 for U14 and their tournament results, as reflected in official ranking lists. The U12 players had a mean training experience of 5.50 ± 0.62 years and trained 6.90 ± 0.90 hours per week, whereas the U14 players had a mean training experience of 7.56 ± 0.78 years and trained 11.10 ± 0.80 hours per week. These training-exposure data are provided descriptively. In addition to regular tennis training, all participants attended mandatory physical education classes (4 × 45 minutes per week). Biological maturity was assessed using the classical five-stage Tanner scale [17]. Tanner stage was determined by participant self-assessment using standardized, realistic illustrations of secondary sexual characteristics developed by Carel and Léger [18]. The assessment was conducted in the presence of a member of the research team who provided instructions and supervised the procedure to ensure correct completion, and in the presence of a parent or legal guardian. This approach has been widely used in studies involving athletic children and adolescents as an ethical and reliable method for estimating biological maturity [19]. The detailed characteristics of the study groups are presented in Table 1.

**Table 1:** Characteristics of the participants.

Variable	U12 (n = 16)	U14 (n = 17)	U	p	r_rb_
Chronological age (years)	11.36 ± 0.82	14.14 ± 0.74	0	<0.001*	1.00
Body height (cm)	153.62 ± 8.75	163.59 ± 6.44	56	0.003*	0.59
Body mass (kg)	43.28 ± 7.25	51.58 ± 7.45	56	0.003*	0.59
Body mass index (kg·m^−2^)	18.25 ± 2.09	19.20 ± 1.84	91	0.110	0.33
Tanner stage (stage)	2 [2 –2]	3 [3 –3]	8	<0.001*	0.94

Data are presented as mean ± standard deviation, except Tanner stage, which is presented as median [interquartile range]. U, Mann–Whitney U statistic; U12, under 12 years of age; U14, under 14 years of age; r_rb_, rank-biserial correlation. *Significant differences at p < 0.01.

Body mass measurements were used for allometric normalization of the HGS test and isometric muscle strength, with the scaling exponents (b) estimated from log-linear regression analyses.

The study was conducted in accordance with the Declaration of Helsinki and was approved by the Bioethics Committee for Clinical Research of the Regional Medical Association in Gdańsk, Poland (approval no. KB-25/20). Written informed consent was obtained from each participant and her legal guardians prior to study enrollment.

### Procedures

#### International tennis number (ITN).

The ITN is a globally recognized tennis‐ranking system that provides an objective measure of a player’s overall playing level. It uses a 10-point scale, ranging from ITN 1 (highest playing standard) to ITN 10 (beginner). The ITN test was conducted on an indoor clay court. Testing was performed in accordance with the International Tennis Federation (ITF) guidelines [20]. A professional coach with 25 years of experience served balls to the players during the test. Scoring followed the predefined ITF criteria for each task (depth, accuracy, volley depth, serve targets, and mobility), which limits subjectivity by relying on observable landing zones and fixed scoring rules. The ITN on-court test yields a point-based total score, calculated as the sum of points obtained across five components: 1) groundstroke depth – 10 alternating forehand and backhand strokes from the baseline (maximum 90 points); 2) groundstroke accuracy – 6 alternating forehands and backhands down the line and 6 cross-court (maximum 84 points); 3) volley depth – 8 alternating forehand and backhand volleys (maximum 72 points); 4) serve – 12 serves, three to each designated target area (maximum 108 points); 5) mobility – a “spider run,” in which the player sprints around the court collecting five balls one at a time and placing them in a designated zone (maximum 76 points). The maximum overall score was 430 points. For every stroke-based task (all except the mobility test), points were awarded for: shot accuracy, based on where the ball landed within the singles court; shot power, with one bonus point if the ball’s second bounce landed between the baseline and the “bonus line” and two bonus points if it landed beyond the bonus line; consistency, with one additional point for every shot executed without error. The resulting ITN total points can be mapped to an ITN skill level according to the official ITF classification [20]. For analyses in this manuscript, we used the point-based outcomes of the ITN on-court test (ITN total points and subcomponent points).

To reduce rater-related variability and standardize ball delivery, the entire ITN protocol was administered by the same coach, with balls delivered using a preprogrammed ball machine (Lobster Grand Elite V LE, Lobster Sports, USA). The test followed the official ITF on-court assessment structure, predefined target zones, and the standardized point system based on ball landing location and bonus lines. All assessments were performed on the same indoor court to minimize environmental variability.

A test–retest reliability check was embedded in the study protocol as a quality-control procedure and completed in a subsample of randomly selected 13 players. The retest was performed 7 days after the initial test to minimize true training-related changes while avoiding same-day fatigue effects, under identical indoor conditions and standardized scoring procedures. Reliability of the point-based ITN outcomes (total points and subcomponent scores) was quantified using the intraclass correlation coefficient (ICC), based on a two-way mixed-effects model with absolute agreement for single measurements (ICC(3,1)), and is presented with 95% confidence intervals (CI).

Test–retest reliability was high across ITN subcomponents and total points. ICC(3,1) values were 0.92 (95% CI 0.75–0.97) for mobility, 0.93 (95% CI 0.80–0.98) for groundstroke depth, 0.97 (95% CI 0.90–0.99) for volley depth, 0.95 (95% CI 0.86–0.99) for groundstroke accuracy, 0.959 (95% CI 0.87–0.99) for serve, and 0.99 for ITN total points.

#### Handgrip strength test.

Maximal isometric HGS was measured using an electronic hand dynamometer with Hercules software (Saehan Corporation, Masanhappo-gu Changwon, Republic of Korea). Each player performed three trials with the dominant hand; the highest value was used for analysis. Participants were seated comfortably with feet flat on the floor and the tested arm supported on a stable, flat surface. The shoulder was in a neutral position, the elbow flexed at 90°, and the wrist maintained at 0–30° of dorsiflexion. Before testing, the dynamometer was calibrated according to the manufacturer’s instructions to ensure measurement accuracy. Immediately prior to each trial, participants were instructed to hold the dynamometer so that the handle fit comfortably—not too tight or loose—grasping it with four fingers around the handle and the thumb positioned on the opposite side [21]. For each attempt, they were asked to squeeze the dynamometer as forcefully as possible for approximately 3–5 s, while receiving standardized verbal encouragement. A 1-minute rest interval between trials prevented muscular fatigue [22]. Results were expressed as absolute values and normalized to body mass.

#### Flexed arm hang test.

The FAH was administered according to the Eurofit Test protocol in an indoor sports hall [23]. A horizontal bar (diameter ≤2.5 cm) was mounted at a height easily reachable by the participants. A protective gymnastics mat was placed underneath, and hand chalk was provided to improve grip. Timing was recorded with a digital stopwatch. Participants grasped the bar using a closed overhand grip at shoulder width, standing on a chair to assume the starting position. At the signal to start, the support was removed and the participant maintained a FAH with the chin held above the bar. The FAH test is a functional field-based measure of upper-body muscular endurance rather than an isolated strength assessment, and performance may be affected by body-position control (sway minimization), as reported for other upper-body suspension tasks [24]. Therefore, participants were carefully instructed to keep the trunk and lower limbs as still as possible and to avoid swinging or kicking movements during the trial. Timing continued until the participant’s chin dropped below the level of the bar. The test was performed once, with time recorded to the nearest 0.01 s. Participants wore light sports clothing.

#### Tennis-ball throw for distance test.

Upper-limb explosive strength was assessed by a TBT test for distance in an indoor sports hall. To eliminate the contribution of the lower limbs, the throw was performed from a half-kneeling position: right-handed players knelt with the left leg forward, and left-handed players with the right leg forward, maintaining an upright trunk. Holding a tennis ball at chest height with the dominant arm, participants executed a maximal forward overhead throw, generating force primarily through the shoulder girdle and trunk while maintaining the kneeling base position. Throw distance was measured in a straight line from the throwing line to the first point of ground contact of the ball, with 1 cm precision. Two official trials were performed after two practice throws; the best distance of the two official attempts was used for analysis. A trial was repeated if the participant lost balance, left the kneeling position, or involved the lower limbs during the throwing motion.

#### Upper limb isometric muscle strength assessment.

Isometric strength testing was performed using a Biodex System 4 isokinetic dynamometer (Biodex Medical Systems, Inc., Shirley, NY, USA). Participants were seated on the dynamometer’s adjustable chair, and the backrest was individually positioned to fit each player’s body in accordance with the manufacturer’s guidelines. To ensure postural stabilization, leather straps were applied across the chest, pelvis, and thigh. Isometric assessments were conducted for three upper-limb joints: glenohumeral, elbow, and radiocarpal joints, each under standardized conditions. For the glenohumeral joint, the tested arm was abducted to 90° in the frontal plane with the elbow flexed to 90°. The hand was in a neutral grip on the dynamometer lever arm, and the device’s axis of rotation was aligned with the olecranon process. In this configuration, the muscle strength of external rotation (GHER) and internal rotation (GHIR) was assessed. For the elbow joint, the shoulder was flexed to 45°, with the entire upper limb supported by a pad beneath the olecranon process. The hand remained in a neutral position, and the dynamometer’s rotational axis was aligned with the lateral epicondyle of the humerus. This position allowed assessment of elbow flexion (EF) and elbow extension (EE) strength. Finally, for the radiocarpal joint, the forearm rested in pronation on a dedicated support, with a broad leather strap around the mid third of the forearm to limit its movement. The forearm and trunk straps were used to stabilize the body and isolate movement to the wrist. The dynamometer lever arm was positioned so that its axis of rotation was aligned with the ulnar styloid process, and the radiocarpal joint was in neutral position between flexion and extension. In this configuration, radiocarpal flexion (RCF) and radiocarpal extension (RCE) strength were evaluated. For each tested action, participants performed three maximal voluntary isometric contractions (MVICs). They were instructed to push or pull the limb segment as forcefully and as rapidly as possible, while receiving strong standardized verbal encouragement to elicit maximal effort. The trial with the highest peak torque (PT) was retained for analysis. For every joint action, the following variables were obtained: PT expressed in newton-meters (Nm), peak rate of torque development (PRTD) expressed in newton-meters per second (Nm·s^−1^). PT was allometrically scaled to body mass, whereas PRTD was normalized to PT, and expressed as Nm·kg^-b^ and Nm·s^−1^·Nm^−1^, respectively.

#### Sense of force testing.

This test was performed using the same dynamometer (Biodex System 4) and the identical joint positions as described in the upper-limb isometric muscle strength assessment. The joints and muscle actions evaluated were likewise the same as those assessed for isometric strength. The procedure consisted of ipsilateral reproduction of a memorized torque level under isometric conditions (torque reproduction test). Using visual feedback, each participant first observed on the dynamometer screen the target torque expressed as a percentage of the previously determined PT during MVIC for the specific joint and muscle action. After a 5-second rest period, she was asked to reproduce the same torque level without any visual or auditory feedback. Two target intensities, 20% and 50% of MVIC, were tested separately for each joint action. For each intensity, three trials were performed. The order of joints, muscle actions and target torque levels was randomized to minimize learning effects and fatigue. For the torque reproduction test, the primary outcome was the absolute error, calculated as the mean of the absolute differences (percentage points) between the target and reproduced torque values across all trials. Lower absolute error values indicated greater accuracy of torque reproduction. The reliability of force-sense assessment with the Biodex system has been previously established. In reference studies, ICC for the torque reproduction test reached 0.81 [25], indicating adequate methodological reliability.

### Statistical analysis

Data are presented as mean ± standard deviation (SD), except for Tanner stage, which is presented as median [interquartile range]. Distributions of continuous variables were assessed using the Shapiro–Wilk test and visual inspection of distributional plots. Because several analysed variables showed departures from normality, between-group comparisons between U12 and U14 players were performed using the Mann–Whitney U test to maintain a consistent non-parametric analytical framework across outcomes. Exact two-sided p-values are reported. Effect sizes for between-group comparisons are presented as rank-biserial correlation (r_rb_), with positive values indicating higher ranks in the U14 group.

For variables reported with allometric scaling, allometric exponents (b) were estimated from the pooled sample using a log-linear model: ln(Y) = a + b· ln(body mass). Allometrically scaled values were calculated as Y· body mass^-b^, where b denotes the outcome-specific allometric exponent indicated in the variable label.

Associations between ITN point-based outcomes, including ITN total points and ITN subcomponent points, and strength-related variables were examined separately in the U12 and U14 groups using Spearman’s rank correlation coefficient (ρ). To control for multiplicity, p-values from correlation analyses were adjusted using the Benjamini–Hochberg false discovery rate procedure. Adjustments were performed separately for each age group and for each ITN outcome across the full panel of strength-related variables. Nominally significant correlations with p < 0.05 are reported in the correlation table; however, only associations with FDR-adjusted q < 0.05 were considered statistically robust. Correlations that reached only nominal significance were interpreted as exploratory and hypothesis-generating, particularly given the age-stratified sample sizes. The magnitude of r_rb_ and ρ were interpreted analogously to correlation coefficients according to Cohen [26] and modified by Hopkins [27] as follows: small (r_rb_/ρ = 0.10–0.29), moderate (r_rb_/ρ = 0.30–0.49), large (r_rb_/ρ = 0.50–0.69), and very large (r_rb_/ρ = 0.70–0.89). These thresholds are commonly used in sport science [28–30].

The level of statistical significance was set at α = 0.05. The required sample size for analysis (n = 26) was estimated a priori using G*Power 3.1.9.4 (Franz Faul, Universität Kiel, Germany) assuming a large effect size for between-group differences (r_rb_ = 0.50) and power of 0.80. Given pragmatic constraints in recruiting competitively trained youth athletes within narrow age categories, the achieved group sizes (U12: n = 16; U14: n = 17) were adequate to detect very large associations (ρ = 0.70) but may have been underpowered for moderate-to-large effects. Therefore, the subgroup-specific correlation analyses should be interpreted as exploratory and hypothesis-generating. All analyses were performed using Statistica 13.3 (TIBCO Software Inc., Palo Alto, CA, USA).

## Results

Across all ITN test components, the U14 female players outperformed the U12 group (Table 2). The mobility assessment was 34.7% higher in U14 (p = 0.002). Scores for groundstroke depth (38.6%, p = 0.002) and volley depth (42.7%, p = 0.017) were also significantly higher in U14 than in U12. In addition, U14 players demonstrated a significantly greater groundstroke accuracy score (54.0%, p = 0.012) and a higher serve score (31.7%, p = 0.017) compared with U12. The ITN total points were 39.4% higher in the U14 group (p = 0.002).

**Table 2:** Tennis performance outcomes derived from the International Tennis Number (ITN) on-court test (mean ± standard deviation).

Variable	U12 (n = 16)	U14 (n = 17)	U	p	r_rb_
Groundstroke depth score	38.19 ± 15.92	52.94 ± 9.15	52.5	0.002**	0.61
Groundstroke accuracy score	32.00 ± 20.06	49.29 ± 17.06	67	0.012*	0.51
Volley depth score	20.00 ± 9.42	28.53 ± 9.27	70.5	0.017*	0.48
Serve score	44.81 ± 17.52	59.00 ± 15.12	70	0.017*	0.49
Mobility assessment score	30.88 ± 9.73	41.59 ± 6.91	51	0.002**	0.62
ITN total points	165.88 ± 59.89	231.35 ± 37.02	51.5	0.002**	0.62

U12, under 12 years of age; U14, under 14 years of age; U, Mann–Whitney U statistic; r_rb_, rank-biserial correlation. Significant differences at p * ≤ 0.05, ** < 0.01.

U14 players demonstrated higher general muscle strength than U12 players (Table 3). Absolute HGS was 28.0% higher (p < 0.001); however, the difference was not statistically significant after allometric normalization to body mass (p = 0.179). FAH duration was 68.2% longer (p = 0.021), and TBT performance was 28.6% higher (p = 0.002) in U14 compared with U12.

**Table 3:** General strength characteristics of the tennis players’ upper limbs (mean ± standard deviation).

Variable	U12 (n = 16)	U14 (n = 17)	U	p	r_rb_
Handgrip strength (kg)	23.74 ± 4.11	30.40 ± 6.16	42	<0.001**	0.69
Handgrip strength/ body mass^b^ (kg ⋅ kg^-0.94^)	0.69 ± 0.09	0.75 ± 0.10	98	0.179	0.28
Flexed arm hang (s)	10.63 ± 8.81	17.87 ± 11.26	72	0.021*	0.47
Tennis ball throw (m)	15.69 ± 3.17	20.18 ± 4.40	52.5	0.002**	0.61

U12, under 12 years of age; U14, under 14 years of age; ^b^, allometric exponent; U, Mann–Whitney U statistic; r_rb_, rank-biserial correlation. Significant differences at p * ≤ 0.05, ** < 0.01.

U14 players exhibited significantly greater absolute PT across upper-limb joint actions compared with the U12 group (Table 4). For GHIR, absolute PT was 31.6% higher in U14 than in U12 players (p = 0.005), whereas PRTD did not differ significantly between groups (p = 0.326). For GHER, absolute PT and PRTD were 21.2% (p = 0.021) and 32.7% (p = 0.049) higher in U14 than in U12 players, respectively. After normalization, between-group differences in glenohumeral PT and PRTD did not reach statistical significance. For EF, U14 players demonstrated a 29.3% higher absolute PT than U12 players (p < 0.001), whereas PRTD did not reach statistical significance (p = 0.058). For EE, absolute PT was 14.4% higher in U14 than in U12 players (p = 0.041), while normalized, it did not differ significantly between groups (p = 0.231). PRTD of EE showed no group differences. All variables of RCF did not differ significantly between groups. In RCE, U14 players showed significantly higher absolute PT by 18.0% (p = 0.041) and PRTD by 41.2% (p = 0.028) compared with U12 players, whereas normalized values were not statistically significant (p = 0.606 and p = 0.068, respectively).

**Table 4:** Upper limb peak torque and peak rate of torque development characteristics of the tennis players (mean ± standard deviation).

Variable	U12 (n = 16)	U14 (n = 17)	U	p	r_rb_
***Glenohumeral internal rotation***					
PT (Nm)	17.98 ± 4.13	23.67 ± 6.44	60.5	0.005**	0.56
PT/ body mass^b^ (Nm·kg^-0.93^)	0.55 ± 0.14	0.60 ± 0.11	88	0.087	0.35
PRTD (Nm·s^−1^)	142.24 ± 48.29	186.18 ± 90.18	108	0.326	0.21
PRTD/ PT (Nm·s^−1^·Nm^-1^)	8.04 ± 2.63	7.75 ± 2.88	126	0.736	−0.07
***Glenohumeral external rotation***					
PT (Nm)	17.30 ± 3.29	20.96 ± 4.90	72	0.021*	0.47
PT/ body mass^b^ (Nm·kg^-0.72^)	1.15 ± 0.20	1.22 ± 0.25	101	0.217	0.26
PRTD (Nm·s ^− 1^)	323.89 ± 115.39	429.83 ± 158.46	81	0.049*	0.40
PRTD/ PT (Nm·s ^− 1^·Nm^−1^)	18.30 ± 4.51	20.28 ± 5.01	101	0.217	0.26
***Elbow flexion***					
PT (Nm)	26.64 ± 5.58	34.44 ± 8.11	47	<0.001**	0.65
PT/ body mass^b^ (Nm·kg^−1.08^)	0.46 ± 0.08	0.48 ± 0.07	108	0.326	0.21
PRTD (Nm·s^−1^)	499.42 ± 130.25	600.82 ± 148.43	83	0.058	0.39
PRTD/ PT (Nm·s^−1^·Nm^−1^)	18.76 ± 2.91	17.67 ± 3.12	111	0.382	−0.18
***Elbow extension***					
PT (Nm)	18.31 ± 6.51	20.95 ± 3.20	79.5	0.041*	0.42
PT/ body mass^b^ (Nm·kg^-0.55^)	2.31 ± 0.79	2.41 ± 0.41	102	0.231	0.25
PRTD (Nm·s^−1^)	170.52 ± 86.00	202.90 ± 83.79	98	0.179	0.28
PRTD/ PT (Nm·s^−1^·Nm^−1^)	9.36 ± 3.69	9.63 ± 3.48	128	0.790	0.06
***Radiocarpal flexion***					
PT (Nm)	7.62 ± 3.52	9.09 ± 3.97	96	0.157	0.29
PT/ body mass^b^ (Nm·kg^−1.14^)	0.10 ± 0.04	0.10 ± 0.04	129	0.817	−0.05
PRTD (Nm·s^−1^)	49.77 ± 25.42	61.18 ± 24.00	91	0.110	0.33
PRTD/ PT (Nm·s^−1^·Nm^−1^)	6.69 ± 1.81	6.87 ± 1.31	123	0.657	0.10
***Radiocarpal extension***					
PT (Nm)	5.16 ± 1.58	6.09 ± 1.57	79.5	0.041*	0.42
PT/ body mass^b^ (Nm·kg^-0.85^)	0.21 ± 0.05	0.21 ± 0.05	121	0.606	0.11
PRTD (Nm·s^-1^)	54.18 ± 30.16	76.48 ± 33.49	75	0.028*	0.45
PRTD/ PT (Nm·s^-1^·Nm^-1^)	10.08 ± 2.66	12.11 ± 3.02	85	0.068	0.38

U12, under 12 years of age; U14, under 14 years of age; PT, peak torque; PRTD, peak rate of torque development; ^b^, allometric exponent; U, Mann–Whitney U statistic; r_rb_, rank-biserial correlation. Significant differences at p * ≤ 0.05, ** < 0.01.

No significant differences were observed between the U12 and U14 female players in torque reproduction test across the tested movements (Table 5).

**Table 5:** Absolute error (percentage points) in torque reproduction test performance of the tennis players (mean ± standard deviation).

Variable	U12 (n = 16)	U14 (n = 17)	U	p	r_rb_
***Glenohumeral internal rotation***					
20% MVIC	4.64 ± 4.53	2.72 ± 1.77	103	0.245	−0.24
50% MVIC	10.30 ± 7.97	10.93 ± 8.57	120	0.581	0.12
***Glenohumeral external rotation***					
20% MVIC	3.82 ± 2.56	3.74 ± 2.47	135	0.986	0.01
50% MVIC	9.75 ± 7.54	6.82 ± 4.85	113	0.423	−0.17
***Elbow flexion***					
20% MVIC	3.27 ± 2.12	2.78 ± 1.75	118	0.533	−0.13
50% MVIC	11.66 ± 7.07	9.67 ± 5.42	115	0.465	−0.15
***Elbow extension***					
20% MVIC	4.13 ± 3.81	3.20 ± 2.30	128	0.790	−0.06
50% MVIC	10.19 ± 6.15	6.69 ± 4.25	85	0.068	−0.38
***Radiocarpal flexion***					
20% MVIC	3.45 ± 2.41	3.08 ± 2.47	118	0.533	−0.13
50% MVIC	8.31 ± 6.07	6.86 ± 3.80	125	0.709	−0.08
***Radiocarpal extension***					
20% MVIC	3.89 ± 3.15	4.82 ± 4.27	120	0.581	0.12
50% MVIC	8.47 ± 4.77	6.39 ± 4.62	99	0.191	−0.27

U12, under 12 years of age; U14, under 14 years of age; MVIC, maximal voluntary isometric contraction; U, Mann–Whitney U statistic; r_rb_, rank-biserial correlation.

Spearman’s correlation analyses between ITN point-based outcomes and strength-related variables are presented in Table 6. After Benjamini–Hochberg false discovery rate correction, two positive associations remained statistically significant: TBT distance with groundstroke accuracy in U12 players (p < 0.001) and PT in EF with ITN total points in U14 players (p < 0.001).

**Table 6:** Spearman’s correlation analysis between ITN point-based outcomes (total points and subcomponent points) and muscle strength variables with Benjamini–Hochberg false discovery rate correction.

Variable		Groundstroke depth		Groundstroke accuracy		Volley depth		Serve		Mobility assessment		ITN total points	
		U12	U14	U12	U14	U12	U14	U12	U14	U12	U14	U12	U14
**HGS**		0.27	0.19	0.38	0.44	0.06	0.08	0.14	0.47	0.32	0.37	0.36	0.50
**HGS/ BM** ^**b**^		0.50	0.00	0.31	0.02	0.26	−0.02	0.37	0.45	0.50	0.39	0.52	0.20
**FAH**		0.40	0.15	0.14	0.32	0.39	0.13	0.35	0.08	0.39	0.67	0.36	0.38
**TBT**		0.54	0.43	0.74*	0.43	0.27	0.13	0.25	0.03	0.55	0.39	0.61	0.36
**GHIR**	**PT**	0.19	0.17	0.02	0.42	0.21	0.36	0.06	0.30	0.14	0.40	0.19	0.58
	**PT/ BM** ^**b**^	0.20	0.20	−0.11	0.14	0.38	0.30	0.13	0.12	0.15	0.52	0.18	0.34
	**PRTD**	0.18	0.14	0.36	0.34	0.21	0.50	0.08	0.32	0.29	0.35	0.28	0.50
	**PRTD/ PT**	0.00	0.01	0.24	0.07	0.01	0.58	0.08	0.14	0.14	0.13	0.10	0.25
	**TR 20% MVIC**	−0.18	0.11	−0.10	−0.13	−0.39	0.12	−0.25	−0.54	0.13	0.06	−0.23	−0.31
	**TR 50% MVIC**	0.08	0.22	0.04	0.09	−0.55*	0.30	0.07	0.26	−0.10	0.01	−0.03	0.31
**GHER**	**PT**	0.00	0.03	−0.07	0.02	0.15	0.26	0.08	−0.00	−0.00	0.08	0.04	0.05
	**PT/ BM** ^**b**^	0.16	−0.18	−0.20	−0.39	0.49	0.19	0.34	−0.08	−0.06	−0.05	0.16	−0.25
	**PRTD**	−0.04	0.36	0.05	0.37	−0.05	0.22	−0.13	0.33	−0.16	0.17	0.00	0.38
	**PRTD/ PT**	−0.09	0.34	0.11	0.52	−0.07	0.10	−0.26	0.49	−0.22	0.05	−0.04	0.49
	**TR 20% MVIC**	−0.14	−0.05	−0.43	0.07	−0.17	−0.25	0.10	0.19	−0.05	−0.28	−0.21	0.06
	**TR 50% MVIC**	0.06	0.02	−0.14	0.27	−0.29	0.21	0.09	0.00	0.07	0.34	−0.04	0.33
**EF**	**PT**	0.28	0.40	0.19	0.53	0.09	0.34	0.19	0.28	0.35	0.48	0.28	0.73*
	**PT/ BM** ^**b**^	0.49	0.33	0.15	0.23	0.40	0.34	0.40	0.24	0.30	0.41	0.44	0.55
	**PRTD**	0.04	0.08	−0.02	0.42	−0.11	0.06	−0.02	0.33	0.06	0.13	−0.02	0.41
	**PRTD/ PT**	−0.11	−0.23	−0.05	−0.09	−0.11	−0.27	−0.21	−0.27	−0.03	−0.15	−0.19	−0.40
	**TR 20% MVIC**	−0.06	0.46	−0.03	0.40	0.04	0.33	−0.16	0.02	0.16	0.69	−0.10	0.42
	**TR 50% MVIC**	0.07	0.07	−0.01	−0.12	−0.40	0.33	0.04	−0.11	0.23	0.07	0.00	0.13
**EE**	**PT**	0.37	0.61	0.29	0.22	0.02	0.32	0.16	0.08	0.60	0.28	0.34	0.38
	**PT/ BM** ^**b**^	0.35	0.34	0.11	−0.18	0.11	0.13	0.23	−0.24	0.61	0.14	0.29	−0.15
	**PRTD**	0.08	0.12	0.18	0.06	0.03	0.42	0.22	0.19	0.07	0.21	0.18	0.22
	**PRTD/ PT**	−0.04	−0.19	0.05	0.09	−0.03	0.30	0.15	0.44	−0.35	0.09	0.06	0.22
	**TR 20% MVIC**	−0.07	0.10	−0.33	0.30	−0.17	0.05	0.19	0.35	−0.09	0.12	−0.15	0.34
	**TR 50% MVIC**	0.05	−0.13	0.04	−0.04	−0.51	−0.29	−0.04	0.04	−0.07	−0.19	−0.04	−0.16
**RCF**	**PT**	0.22	0.25	0.17	0.05	0.10	0.42	0.19	0.09	0.32	0.32	0.25	0.32
	**PT/ BM** ^**b**^	0.25	0.39	0.08	−0.02	0.22	0.32	0.33	−0.17	0.36	0.37	0.28	0.16
	**PRTD**	0.25	0.38	0.41	0.30	−0.11	0.33	0.09	0.15	0.19	0.53	0.26	0.45
	**PRTD/ PT**	0.04	−0.28	0.33	0.31	−0.03	−0.15	−0.10	0.34	−0.26	0.26	0.06	0.17
	**TR 20% MVIC**	0.07	−0.40	0.33	−0.60	−0.22	0.24	−0.01	−0.25	0.24	−0.33	0.06	−0.37
	**TR 50% MVIC**	0.00	−0.16	0.04	0.04	−0.28	−0.04	−0.09	0.36	0.05	−0.33	−0.06	−0.02
**RCE**	**PT**	0.24	0.26	0.34	0.47	−0.11	−0.06	−0.09	−0.16	0.37	0.23	0.23	0.34
	**PT/ BM** ^**b**^	0.47	0.18	0.42	0.23	0.08	−0.19	0.08	−0.43	0.46	0.29	0.40	0.08
	**PRTD**	0.24	0.54	0.35	0.59	−0.21	−0.18	−0.15	−0.17	0.30	0.46	0.16	0.46
	**PRTD/ PT**	0.21	0.56	0.15	0.50	−0.28	−0.18	0.05	−0.14	0.08	0.62	0.10	0.45
	**TR 20% MVIC**	0.12	0.24	−0.02	0.10	0.05	−0.01	0.19	0.13	0.01	0.06	0.09	0.19
	**TR 50% MVIC**	0.17	0.32	0.32	0.16	0.06	−0.06	0.24	0.09	0.04	0.15	0.28	0.25

U12, under 12 years of age; U14, under 14 years of age; HGS, Handgrip strength; FAH, Flexed arm hang; TBT, Tennis ball throw; MVIC, maximal voluntary isometric contraction; PT, peak torque; BM, body mass; ^b^, allometric exponent; PRTD, peak rate of torque development; TR, torque reproduction; GHIR, glenohumeral internal rotation; GHER, glenohumeral external rotation; EF, elbow flexion; EE, elbow extension; RCF, radiocarpal flexion; RCE, radiocarpal extension. Values are Spearman’s rank correlation coefficients (ρ). Nominal p < 0.05 are shaded gray; *FDR-adjusted q < 0.05 after Benjamini–Hochberg correction applied separately for each ITN outcome within each age group.

Additional associations reached nominal significance based on uncorrected p-values (p ≤ 0.05), but did not remain statistically significant after FDR correction. In U12 players, these associations were observed mainly for general strength variables: TBT distance correlated with groundstroke depth, mobility assessment, and ITN total points (p = 0.030, p = 0.027, and p = 0.012, respectively), while HGS normalized to body mass^b^ correlated with groundstroke depth, mobility assessment, and ITN total points (p = 0.049, p = 0.047, and p = 0.040, respectively). Mobility assessment was also correlated with PT and PT normalized to body mass^b^ in EE (p = 0.013 and p = 0.012, respectively). Negative nominally significant correlations were observed between volley depth and torque reproduction error at 50% MVIC for GHIR and EE (p = 0.026 and p = 0.044, respectively).

In U14 players, nominally significant associations were observed more frequently for joint-specific strength and rapid torque-production variables. ITN total points correlated with HGS, PT and PRTD in GHIR, normalized PRTD in GHER, and PT normalized to body mass^b^ in EF (p = 0.041–0.014). Groundstroke depth and accuracy were associated mainly with PT in EF and EE and PRTD-related variables in RCE (p = 0.009–0.042). Volley depth correlated with PRTD-related variables in GHIR (p = 0.015–0.043), whereas serve score correlated positively with normalized PRTD in GHER (p = 0.045) and negatively with GHIR torque reproduction error at 20% MVIC (p = 0.024). Mobility assessment correlated with FAH, PT normalized to body mass^b^ in GHIR, torque reproduction error at 20% MVIC in EF, PRTD in RCF, and normalized PRTD in RCE (p = 0.003–0.031).

## Discussion

The aim of the present study was to compare multiple aspects of muscle strength between U12 and U14 female tennis players in relation to their ITN scores in terms of ITN total points and ITN subcomponent points.

### Tennis performance

In this study U14 female players showed significantly higher ITN point-based scores than U12 players, which is consistent with higher on-court performance in the older age category and with their longer playing experience [31,32]. Previous studies suggest that important age-related differences in competitive tennis skills may be observed between 10 and 14 years of age, a period often considered relevant for training planning and talent identification [7,31,32]. The ITN levels (ordinal ratings) in our cohort (U12: 6.75; U14: 4.59) indicate a relatively high standard of competitive play, as players aged 13–14 years typically achieve ITN scores of approximately 3–6 depending on competitive level and training intensity [7,31,33]. ITN is a valuable tool for monitoring players’ progress and planning individualized training [1], yet few studies have investigated its relationship with upper-limb muscle strength in young female athletes [34].

Adequate muscular strength may support the execution of several technical skills, including strokes requiring depth, spin, or postural stability. Therefore, strength-related capacities may be relevant to tennis performance, although their role should be interpreted alongside technical, tactical, and developmental factors [3,35,36]. Previous research indicates that systematic increases in training load during long-term training may facilitate strength development, improve on-court movement, and positively affect ITN scores [34]. In the present cross-sectional sample, U14 players had greater training exposure and higher ITN point-based scores than U12 players, but these data do not establish within-player training-related change.

### General muscle strength

U14 players demonstrated significantly greater general muscle strength than U12 players, a difference mainly explained by their larger muscle mass after body-mass^b^ normalization of HGS [37]. Pubertal development differed between age categories, as indicated by higher Tanner stage in U14 compared with U12. Therefore, between-group differences in strength and ITN outcomes likely reflect a combined influence of chronological age, biological maturation, and accumulated training exposure. Comparable age-related increases have been observed in girls aged 11–16 years [38]. While Elliott et al. [38] found that higher tennis performance consistently coincided with greater overall strength, Ulbricht et al. [39] suggested that this link may be confined to U12 and U16 players, but not to U14. This indicates the possibility of differentiated relationships between strength and tennis performance in the U12 and U14 groups. In the correlation analysis, the strongest and FDR-robust association in U12 players was observed between TBT distance and groundstroke accuracy. Additional nominal associations in U12 involved TBT distance and normalized HGS with selected ITN outcomes, particularly groundstroke depth, mobility assessment, and ITN total points. In U14 players, general strength variables showed fewer nominal associations with ITN outcomes; HGS was nominally associated with ITN total points, whereas FAH was nominally associated with mobility assessment. Thus, the present data provide stronger support for a relationship between general upper-limb strength and selected ITN components in U12 players than in U14 players. These findings are broadly consistent with previous studies [40,41], which highlight associations between HGS and tennis-specific performance in young players.

The relationship between the results of general muscle strength tests and the ITN components (primarily groundstroke) revealed exclusively positive correlations in the U12 group. This may be related to the fact that players in the U12 category are still characterized by a relatively low level of muscle strength at this age, which depends mainly on body mass (normalized HGS) and is also evident when they do not have to overcome large external resistances (TBT test) [42]. The above findings appear to be supported by the positive correlations observed between the aforementioned muscle strength tests and the mobility assessment (exclusively in the U12 group), which primarily involves body displacement in an agility evaluation task. This is consistent with previous research [39], where in tests not normalized by body mass (2-kg medicine ball throw), the effect size of correlations with tennis performance increased with age. Considering the ITN total points, positive correlations with absolute HGS were again observed in the U14 group, while normalized values were associated in the U12 group. These results are consistent with other measures of tennis performance in female players aged 12–16, such as ranking position, where higher HGS test scores were linked to better ranking outcomes [39]. It should be noted that ITN (r = 0.55–0.63) demonstrates a stronger association with muscle strength measured in this way compared to ranking (r = 0.19–0.30).

### Joint-specific upper-limb strength

U14 players showed higher absolute joint-specific isometric strength than U12 players in most upper-limb actions, although these differences were largely attenuated after body-mass normalization. U14 players showed higher values than U12 players in most joint actions except for RCF and normalization indicated that these differences were mainly due to greater body mass. This pattern agrees with reports of a pronounced age-related increase in upper-limb muscle strength, particularly in the shoulder girdle and elbow flexors, associated with body-mass gains in young tennis players and other overhead athletes [43–45]. These findings suggest that higher absolute strength in U14 players was observed in upper-limb segments functionally involved in tennis strokes, especially those contributing to shot production. Tennis strokes, by contrast, are dynamic, multi-joint actions characterized by high angular velocities and proximal-to-distal energy transfer. This methodological distinction limits direct inference from the present laboratory measures to racket-head speed or ball velocity. Recent biomechanical studies using combined kinematic and sEMG approaches further support this distinction by demonstrating integrated kinetic-chain coordination during tennis serving [46]. Nevertheless, these variables remain functionally relevant as neuromuscular capacity indicators related to upper-limb control and force transfer during stroke execution [47].

Between-group differences in PRTD were less consistent than those observed for absolute PT. Significantly higher PRTD in U14 players was observed for GHER and RCE, whereas EF did not reach statistical significance (p = 0.058), and no significant differences were found for GHIR, EE, or RCF. This suggests that age-related differences in rapid torque production were not generalized across all upper-limb joint actions. The higher PRTD in RCE observed in U14 players may reflect differences in neuromuscular characteristics and training exposure, including higher initial motor-unit discharge rates, improved synchronization, reduced electromechanical delay, and refined intermuscular coordination [45,47–49]. These neuromuscular characteristics may help explain how strength-related capacity could translate into on-court performance under the present testing framework. Repeated stroke patterns at the shoulder and elbow acceleration contribute to racket speed and ball spin [50,51].

Analysis of the relationships between isometric joint strength and ITN point-based outcomes indicated that only one joint-specific association remained statistically significant after FDR correction: PT in EF with ITN total points in U14 players. Additional nominally significant associations in U14 were observed for selected glenohumeral, elbow, and radiocarpal variables, particularly PT and PRTD in GHIR, normalized PRTD in GHER, PT in EF and EE, and RCE PRTD-related variables. In U12 players, joint-specific associations were less frequent and were mainly limited to absolute and normalized PT in EE in relation to mobility assessment. These nominal patterns suggest that joint-specific strength capacity may become more relevant to ITN point-based performance in older players; however, this interpretation should remain cautious because most associations did not survive multiplicity correction.

These results suggest an association between greater joint-specific strength capacity and higher point-based performance in selected ITN components; however, interpretation should be cautious in a cross-sectional framework. Such relationships are consistent with the biomechanical model of the tennis stroke, in which both precise control of glenohumeral rotations and rapid acceleration of the distal segment of the upper limb are critical [47,52–54]. This is particularly evident in the long-term development of glenohumeral muscle strength, where GHIR progressively exceeds that of the antagonists [55–57]. While this adaptation reflects the progressive refinement of tennis performance, it simultaneously increases the risk of shoulder injury [43,58–60]. Targeted strengthening of selected shoulder muscle groups, particularly the glenohumeral external rotators, has been suggested as a strategy to optimize muscle strength and the balance between the internal and external rotators [61,62]. Such interventions may enhance dynamic shoulder stability and neuromuscular control and potentially reduce the risk of injuries in overhead athletes, including tennis players [63–65]. Moreover, radiocarpal- and elbow-related differences support the value of monitoring the flexor–extensor complex in young overhead athletes, as neuromuscular imbalance or reduced load tolerance around the joints may contribute to overuse-related conditions such as lateral epicondylitis [66,67]. In athletes with chronic lateral epicondylitis, eccentric isokinetic protocols targeting elbow function have been shown to improve neuromuscular and functional outcomes [68].

Most studies examining the relationship between joint muscle strength and tennis performance have focused on serve velocity, yet findings remain inconsistent: some report significant positive correlations with glenohumeral strength [35,53,69], whereas others do not [34]. In the present study, although several upper-limb strength measures correlated with ITN components, serve performance itself showed no relationship with PT. This is not surprising given that serve velocity is typically assessed independently of the accuracy that contributes to the ITN score. By contrast, GHER normalized PRTD showed a nominally positive association with the serve component of ITN, suggesting that rapid torque-production capacity relative to PT may contribute to serve effectiveness. However, this association did not remain statistically significant after FDR correction and should therefore be interpreted cautiously [51].. This pattern differs from [53], who reported strong associations between internal rotation strength and serve velocity for both PT and PRTD. Participants in that study (mean age 14.1 ± 1.2 years) had a higher ITN level (3), included both sexes, and served without the accuracy element considered in ITN scoring. These differences highlight that ITN on-court test outcomes should be interpreted not only as a final composite score but also with attention to the performance of its individual components.

One of the few studies linking joint-specific muscle strength with the ITN is that of [34], who found a positive correlation (r = 0.58) between isometric RCF and tennis performance. In the present study, PT of RCF showed no association with ITN total points after multiplicity correction. Instead, nominally significant associations were observed mainly for RCE in PRTD-related variables with groundstroke depth, groundstroke accuracy, and mobility assessment in U14 players. It should be noted that [34] investigated boys who were older (17.0 ± 0.8 years) than the U14 category. This pattern may indicate a role of rapid wrist-extension torque capacity in selected ITN components, although these findings should be treated as preliminary. The above observation is in line with the findings of Dobos et al. [70], who reported a strong positive relationship (r = 0.86) between rate of force development of the forearm muscles and post-impact ball velocity during the flat serve. Together, these findings suggest that rapid torque-production capacity at the wrist may be relevant to technically demanding tennis actions, but the present data do not support a robust association between RCF strength and overall ITN performance.

### Sense of force

Torque reproduction provides a joint-specific measure of force sense, a proprioceptive function involved in motor control [71,72]. No significant differences in torque reproduction were observed between U12 and U14 players across any tested joints or functions. For EE at 50% of MVIC, the between-group difference was also not statistically significant (p = 0.068). Previous studies indicate that upper-limb proprioceptive accuracy and force sense may vary with age and training experience [73,74]. Studies in athletic populations have shown that better performance in torque reproduction test is typically associated with higher technical proficiency and greater training experience [14]. These results suggest that force-sense, as assessed under the present protocol, may mature before age 12, may differentiate further after age 14, or may not be captured sensitively by the torque reproduction paradigm in this age range. It should be noted that presented torque reproduction provides an index of force-sense under static, joint-specific conditions, whereas tennis-specific stroke control depends on dynamic, multi-segment coordination. Therefore, the present torque reproduction outcomes should not be interpreted as direct evidence of tennis-specific stroke-control proficiency.

The direction of nominal associations between torque reproduction error and ITN outcomes was not uniform. In U12 players, lower torque reproduction error at 50% MVIC for GHIR and EE was nominally associated with higher volley depth score. In U14 players, lower GHIR torque reproduction error at 20% MVIC was nominally associated with higher serve score, and lower RCF torque reproduction error at 20% MVIC was nominally associated with higher groundstroke accuracy. By contrast, EF torque reproduction error at 20% MVIC was positively associated with mobility assessment. These mixed directions suggest that torque reproduction outcomes may partly reflect the torque level from which the reproduction task was derived rather than a direct and consistent relationship between force sense and ITN performance. Higher muscle strength has previously been linked to poorer torque reproduction accuracy, possibly due to the difficulty of estimating force when a larger motor-unit pool is recruited and to strength imbalances that compromise proprioception [75,76]. Notably, in U14 players significant correlations were limited to distal joints whose contribution to stroke generation is secondary to the trunk and shoulder [77–79]. Overall, the sense of force appears to play different roles across age groups and is not a straightforward determinant of tennis performance, which depends on the integrated action of multiple segments of the kinetic chain rather than on the control of a single muscle group.

Overall, the findings indicate that U14 players achieved higher ITN point-based outcomes and higher absolute strength values than U12 players, although many torque differences were diminished after body-mass^b^ normalization. The correlation analysis provided FDR-robust support for an association between TBT distance and groundstroke accuracy in U12 players, and between PT in EF and ITN total points in U14 players. The broader nominal association patterns are compatible with the view that general strength may be more closely linked to selected ITN components in younger players, whereas joint-specific strength and rapid torque-production characteristics may become more relevant in older players. However, these patterns should be interpreted as exploratory and require confirmation in larger, maturity-informed cohorts.

### Limitations

Biological maturation can influence neuromuscular development through hormonal changes and ongoing maturation of the nervous system, and therefore may affect both strength expression and on-court performance. In the present study, pubertal development was assessed using Tanner stage; however, more detailed maturity indices such as maturity offset or age at peak height velocity were not available. Because chronological age and pubertal development were strongly coupled in this cohort, residual confounding by maturation and growth-related factors cannot be fully excluded. Nevertheless, evidence from youth tennis suggests that maturation alone may not fully explain between-group differences in strength when values are normalized to body mass. For example, a study in U13 and U15 female tennis players comparing peri- and post-peak height velocity reported no differences in body-mass-normalized muscle strength [80]. In addition, in young tennis players aged 9–17 years, seasonal changes in neuromuscular performance were reported to be largely unaffected by maturation [81]. Taken together, these observations support cautious interpretation of age-category differences and association patterns in the present cross-sectional sample. Although Tanner stage provides a pragmatic index of pubertal development, we cannot fully disentangle the independent contributions of maturation, growth-related scaling, and training exposure to the observed results. Given the limited overlap in Tanner stage distribution between groups, adjustment models would be difficult to interpret and were not used. Although most indicators of muscle strength were normalized to body mass, the analysis did not include a multivariable model that would simultaneously account for potential confounders such as overall body size, limb length, and leverage, along with training volume and years of playing experience represented by the two age groups.

Although the Benjamini–Hochberg false discovery rate procedure was applied to account for multiplicity, only two associations remained statistically significant after correction. Therefore, correlations reaching only nominal significance based on uncorrected p-values should be interpreted as exploratory rather than confirmatory, particularly given the number of tests and the age-stratified sample sizes. Replication in larger cohorts is required to confirm the robustness and generalizability of these findings. A further point to consider is how tennis performance and strength variables were operationalized. Tennis performance was assessed using the ITN on-court evaluation, which emphasizes consistency, depth, accuracy, and mobility under standardized, coach-fed conditions. This approach does not fully capture match play demands such as tactical decision-making, adaptation to an unpredictable opponent, or isolated ball velocity. Moreover, although isometric, fixed-angle assessments of upper-limb muscle strength and force sense are widely used in research [35,45,82], they have limited ecological validity, as they do not fully represent the dynamic, multi-joint, and high-velocity nature of tennis strokes; recent work using multi-joint isokinetic profiling and principal component analysis indicates that combined joint-strength patterns may better explain global athletic performance than isolated joint measures [83]. Consequently, the findings should be interpreted as reflecting general neuromuscular and proprioceptive capacities rather than sport-specific performance.

Finally, the players included in the present study were competitively trained, preselected young female athletes rather than a recreational or unselected junior population. Consequently, the applicability of these results is primarily limited to similarly trained female players of comparable competitive level and may not extend to boys or to less experienced athletes.

## Conclusions

In this cohort of competitively trained young female tennis players, U14 athletes achieved higher ITN total points and subcomponent points than U12 athletes, indicating superior on-court performance in the older age category. The U14 group also showed higher values in most general strength tests and in joint-specific upper-limb strength and rapid torque production variables. However, after body-mass^b^ normalization, all between-group differences in PT were no longer statistically significant, suggesting a substantial contribution of greater body size in the older players. No consistent differences were observed between groups in force reproduction accuracy.

Regarding the association analyses, after correction for multiple comparisons, two associations remained statistically significant: tennis ball throw distance with groundstroke accuracy in U12 players and elbow flexion PT with ITN total points in U14 players. Other correlations did not remain statistically significant after FDR correction and should therefore be considered exploratory.

Overall, the findings suggest possible age-category-specific relationships between selected strength characteristics and ITN point-based performance in young female tennis players. Given the cross-sectional design, age-stratified sample sizes, and extensive correlation testing, these results require confirmation in larger, maturity-informed cohorts before they can be translated into prescriptive training recommendations.

## Supporting information

S1 File Raw dataset. (XLSX)
